# Carcinoma of the Stomach, Bowels and Omentum

**Published:** 1874-07

**Authors:** K. P. Moore

**Affiliations:** Knoxville, Ga.


### CARCINOMA OF THE STOMACH, BOWELS AND OMENTUM.

By K. P. MOORE, M.D., of Knoxville, Ga.

*Read before the South-Western Georgia Medical Association, at Fort Valley, May 6, 1874, and published by request of the Society.*

A. J. P., age about sixty, farmer. Applied for treatment about July 1, 1873. Complaining of pains in the back, usually brought on and aggravated by certain movements of the body. There was some little constipation of bowels, but otherwise no disturbance of sufficient character to attract any special notice. Knowing that the business engagements of my patient—those of Sheriff of the county—had kept him on horse-back most of the time for some weeks, I simply directed rest, and gave him, a few compound cathartic pills, with directions to keep the bowels in soluble condition. He improved considerably for several days. Purgatives always relieved his pains. August 4th, complains more than before; pains in the back about the renal region; states that for several months he has been gradually losing strength and flesh.

I was now impressed with the idea that there was something more serious the matter with my patient, and, upon inquiry into previous history, disclosed the following facts: In 1832, while a young man, he was suddenly seized with excruciating pain in left iliac and inguinal region, which lasted for several hours, and passed off. A few days later he voided a stone from the bladder. Some weeks subsequently he had a similar attack, but made a good recovery as before. For twenty years he was now exempt from suffering. About the year 1854 he had a similar attack, from which he made a bad recovery; was confined to his bed for several weeks; the exact nature of the cause I was unable to ascertain. Since that time health not good as before, and for the past several years declined pretty fast. Physical exploration now revealed a vague, irregular enlargement in left iliac region, with distinct dullness on percussion, and more or less tenderness. Urine also examined, and found to throw down upon cooling a muco-semi-floculent deposit.

Diagnosis Nephritic trouble of obscure character. Treatment. Infusion of Buchu leaves, to be used freely Tr. Mur. Iron gtt. xxv. *ter in die.* Brom. Pot., Hydrate Chloral, each grs. xij. at night to procure rest. Patient urged to leave off official duties, and give himself up to rest.

For some weeks the patient improved rapidly, so much so as to resume the duties of his office. Some time in the early part of September he again became worse, and Dr. Harwood was invited to see him. The iliac enlargement was now prominent and quite tender, with much dullness. Except the occasional paroxysms of pain, and the loss of strength, there seemed but little disturbance. Appetite, digestion and sense of taste all natural; bowels a little inclined to constipation; urinary organs performing their function well. The urine submitted to heat and carbolic acid revealed more or less albumen; the deposit upon cooling now presented the appearance of muco-gelatin, and somewhat floculent. The enlargement in the iliac, or, I might more properly say, umbilico-iliac region, appeared to be somewhat pulsating, and gave the peculiar bruit or souffle characteristic of aneurism.

These questions presented themselves to our minds: Could it be aneurism, or was it a case of morbus brightii, or calculus within the pelvis of the kidney? No positive conclusion was arrived at. He continued to go downward in spite of my best efforts. Various prescriptions were tried, but to no permanent good.

October 6th. He went with me to Fort Valley, where we met Drs. Mathews, Green, Jones, Ross, Holt, Mérrett, and perhaps others. The result of which consultation the most of you are familiar with. No positive diagnosis was made out, some believing that his condition was attributable to fâtigue and the wear of active life; others leaning strongly towards renal calculus; some suspecting bright's disease; and others looking to the liver as well as the kidneys; but none suspecting the real trouble.

Acting upon the suggestion of Dr. Mathews, who, by request of the patient, was the principal consulting physician, in regard to treatment, I returned to simple diuretics and tonics, urging the importance of rest, good nourishment, etc. To cut a long story short, the patient grew rapidly worse from this time; enjoying now and then a little lighting up of the taper of life; lifting him now upon the wings of hope, now dropping him low into the darkness of despair. In his last days he was troubled greatly with nausea, suffering a great deal, until, on the night of February 8th, 1874, he found relief in death.

Autopsy twelve hours after death. Rigor mortis only partial. Examination limited by family to abdominal viscera. There was in the abdominal cavity about four gallons of thick, yellow serum, more or less mixed and thickened with excretions from the ragged, ulcerating, cancerous surfaces, which were found here and there throughout the whole cavity. Lying just beneath the stomach, and for its whole length, was a considerable ulcerating, cancerous growth. The greater omentum was a solid mass of scirrhus, with here and there an ulcerating patch. The lesser omentum was thickly studded with scirrhus nodules, as was also the peritoneal covering of the intestines. Here and there on the abdominal walls were stray ulcerating patches. The liver was also the seat, more or less, of cancerous nodules, none ulcerating. The kidneys were the least implicated of any of the viscera examined. They seemed exempt from cancer, and the only material alterations in their structure were two or three little unaccountable blisters, under their thin outer covering, holding one or two teaspoonsful of clear liquor. Upon opening these, depressions corresponding in size were found. On the descending colon, and near its junction with the rectum, on the inner side, was a considerable pouch, the size of fœtal head at term. The walls of the colon forming this pouch were quite thin, attenuated and almost transparent; it was filled with air.

One of the points of interest in this case was the absence of any symptoms referable to the true condition which existed, and the prominent symptoms leading to the suspicion of renal trouble. The urine always gave abundant evidence of albumen. In the latter weeks there was nausea, anasarca, and general œdema, all prominent symptoms of bright's disease of the kidney. There were mucous and muco-gelatinous deposits in great abundance, pains in renal region, and a gravely diathesis, all strongly pointing to probable renal calculus, but little to cause even suspicion of carcinoma. Had I been denied the privilege of post-mortem,

I should always have believed that my patient died either of Bright's kidney or renal calculus.

I quote one or two passages from Watson's Practice. He says: "Carcinoma of the stomach has sometimes no symytoms at all, or none which the most sagacious practitioner would refer to the organ affected. Not long since," says he, "I saw, in consultation, an elderly clergyman, who complained of pains in his back, aggravated by movements of his body. His bowels were costive, and purgatives always relieved his pains. He was passing lithic acid gravel. The pains were felt in or near the renal region. Several years before he had suffered in a similar manner, and had then been cured by being cupped on the loins. What was the matter here? Was it lumbago? Was it a calculus in one of his kidneys? These were the best guesses I could make. The eminent physician whom I met, and a surgeon of no less eminence, who had seen him previously, had not been able to attain any more exact diagnosis. Upon this gentleman's death, which occurred not long afterwards, his disorder was discovered to have been cancer of the stomach."

Again he says: "A young woman came into Middlesex Hospital, under one of my colleagues, with a pulsating tumor in her epigastrium. It was thought at first to be an aneurism, and on that account attracted much attention; but the tumor subsided very much after free purgation. This led some to conclude that it was formed by accumulated fæces in the transverse colon. There was no sickness, nor indeed any one symptom referable to the stomach. She died. The tumor was cancerous and in the stomach. Other similar cases are given by Wood, and cases to the same effect are related by Dr. Seymour in the *Medico-Chirurgical Transactions*, and by M. Andral in his *Clinique Medicale*."

